# The contralateral organization of the human nervous system as a quantum unfolded, holographic-like, artifactual representation of the underlying dynamics of a fundamentally two-dimensional universe

**DOI:** 10.3389/fnsys.2023.987086

**Published:** 2023-05-31

**Authors:** Ronald L. Zukauskis

**Affiliations:** Soliant Health, Atlanta, GA, United States

**Keywords:** multisensory/cross-modal processing, perception, perceptual organization, proprioception, kinesthesis, sensory plasticity/adaptation, spatial cognition

## Abstract

A working hypothesis is put forward in this article that the contralateral organization of the human nervous system appears to function like a quantum unfolded holographic apparatus by appearing to invert and reverse quantum unfolded visual and non-visual spatial information. As such, the three-dimensional contralateral organization would be an artifactual representation of the underlying dynamics of a fundamentally two-dimensional universe. According to the holographic principle, nothing that is experienced as three-dimensional could have been processed in a three-dimensional brain. Everything we would experience at a two-dimensional level would appear as a three-dimensional holographic representation, including the architecture of our brains. Various research observations reported elsewhere are reviewed and interpreted here as they may be related in a process that is fundamental to the underlying two-dimensional dynamics of the contralateral organization. The classic holographic method and characteristics of image formation contained by a holograph are described as they relate to the working hypothesis. The double-slit experiment is described and its relevance to the working hypothesis.

## Introduction

An increasing number of physicists now believe the three-dimensional world we experience is brought about by a kind of digital holographic system; in which information is folded (i.e., stored two-dimensionally) in quantum waves that when unfolded produce conscious visual and non-visual spatial appearances that appear to be within a holographically three-dimensional human nervous system.

The idea that the universe we experience is a hologram has been called “the holographic principle”. The holographic principle posits the universe around us, which we are used to thinking of as being three-dimensional space is actually at a fundamental level two-dimensional; so, everything we see going on around us in three dimensions is happening in a two-dimensional plane. Susskind (2008) asserts, “The three-dimensional world of ordinary experience—the universe filled with galaxies, stars, planets, houses, boulders, and people—is a hologram, an image of reality coded on a distant two-dimensional surface.” When physicists assume the holographic principle is true for doing mathematical calculations, ponderous physics problems—such as the reconciling of gravity and quantum mechanics and the nature of black holes—become much simpler to solve. The laws of physics appear to make more sense when written in two dimensions than in three. While these calculations do not provide direct evidence for the universe being a three-dimensional hologram, they offer intriguing suggestions that it could be (Susskind, 2008; Headrick, 2010; Headrick et al., 2014).

Analogous to the digital informational bits and bytes on a compact disc, which encode sound and motion pictures, the digital matter particles/informational units that make up the two-dimensional universe are encoded in quantum waves (Susskind, 1995; Bekenstein, 2003; Bousso, 2003; Headrick, 2010; Headrick et al., 2014). A wave interference pattern results when two or more waves, such as water waves, crisscross and ripple through each other. Any wave-like phenomena, such as light and radio waves, can create an interference pattern. Quantum waves can be thought of as wave interference patterns that combine alternating regions of constructive and destructive matter particles; which can be encoded to include an infinite number of intermediate interference patterns generated by two or more waves, encoding all the infinite real numbers included between 0 and 1. A quantum bit (qubit), unlike a classical bit on a compact disc which is either 1 or 0, provides many more choices. An infinite valued logic such as fuzzy logic can be used in this regard. It has been shown that fuzzy logic is related to Bayesian Probabilistic Inference and Quantum Logic (Gentili, 2021). Human behavior is highly consistent with Bayesian Probabilistic Inference in both the sensory and motor and cognitive domains (Pouget et al., 2013).

Hypotheses have been made that link the human nervous system and quantum mechanics. Hameroff et al. (2014) have proposed that the operation of consciousness is associated with an explicit wave function collapse that occurs at the level of Microtubles (MTs), which act as quantum computers that process quantum bits. A major question about the MTs potential for quantum information processing is: “How can delicate quantum states, which in the technological realm demand extreme cold and isolation to avoid environmental ‘decoherence,’ manage to survive in the warm, wet brain” (Woolf and Hameroff, 2001). Additionally, Thaheld (2008) concluded there would be no possibility for quantum state reduction in the brain as Hameroff et al. hypothesize because the quantum state of photons collapses in the retina and does not reach the rest of the brain. However, Salari et al. (2008) proposed a quantum teleportation mechanism between the retina and the rest of the brain, which would allow external quantum information to reach throughout the brain. Baars and Edelman (2012) note that about half the human brain does not empirically support conscious activity, yet neurons in these brain areas contain large numbers of MTs. Tarlaci and Pregnolato (2016) review quantum brain concepts and provide 158 references. Of relevance to this article, they discuss the unsolved role of the “observer” in quantum physics; i.e., whether the observer is merely an observer or a “participant” in determining the outcome of a quantum measurement.

A working hypothesis is put forward in this article that the contralateral organization of the human nervous system appears to function like a quantum unfolded holographic apparatus by appearing to invert and reverse quantum unfolded visual and non-visual spatial information. As such, the three-dimensional contralateral organization would be an artifactual representation of the underlying dynamics of a fundamentally two-dimensional universe. If the holographic principle is correct, nothing that is experienced as three-dimensional could have been processed in a three-dimensional brain. Everything we would experience at a two-dimensional level would appear as a three-dimensional holographic representation, including the architecture of our brains. Thus, whether or not a warm, wet brain would affect MTs potential for wave function collapse may not be relevant in a two-dimensional plane where digital matter particles/informational units are encoded in quantum waves, which require no wave function collapse because they are collapsed (i.e., folded). Moreover, a quantum teleportation mechanism between the retina and the rest of the brain would not seem relevant because photons could not collapse in a three-dimensional holographic retina. Furthermore, the three-dimensional holographic representation we experience, although appearing to be unbelievably accurate, would be incomplete in comparison with what may be an infinitely, or near infinitely, accurate two-dimensional source; because such a transformation could never be as accurate as the totality it originated from. For this reason, anomalies would show up that ignore laws that appear to rule the three-dimensional holographic representation. Even so, an unmistakable pattern of up-down inverted and right-left reversed properties do appear that seem integral to the perceived operations of holographic devices, quantum waves, and the contralateral organization.

**Figure 1:**
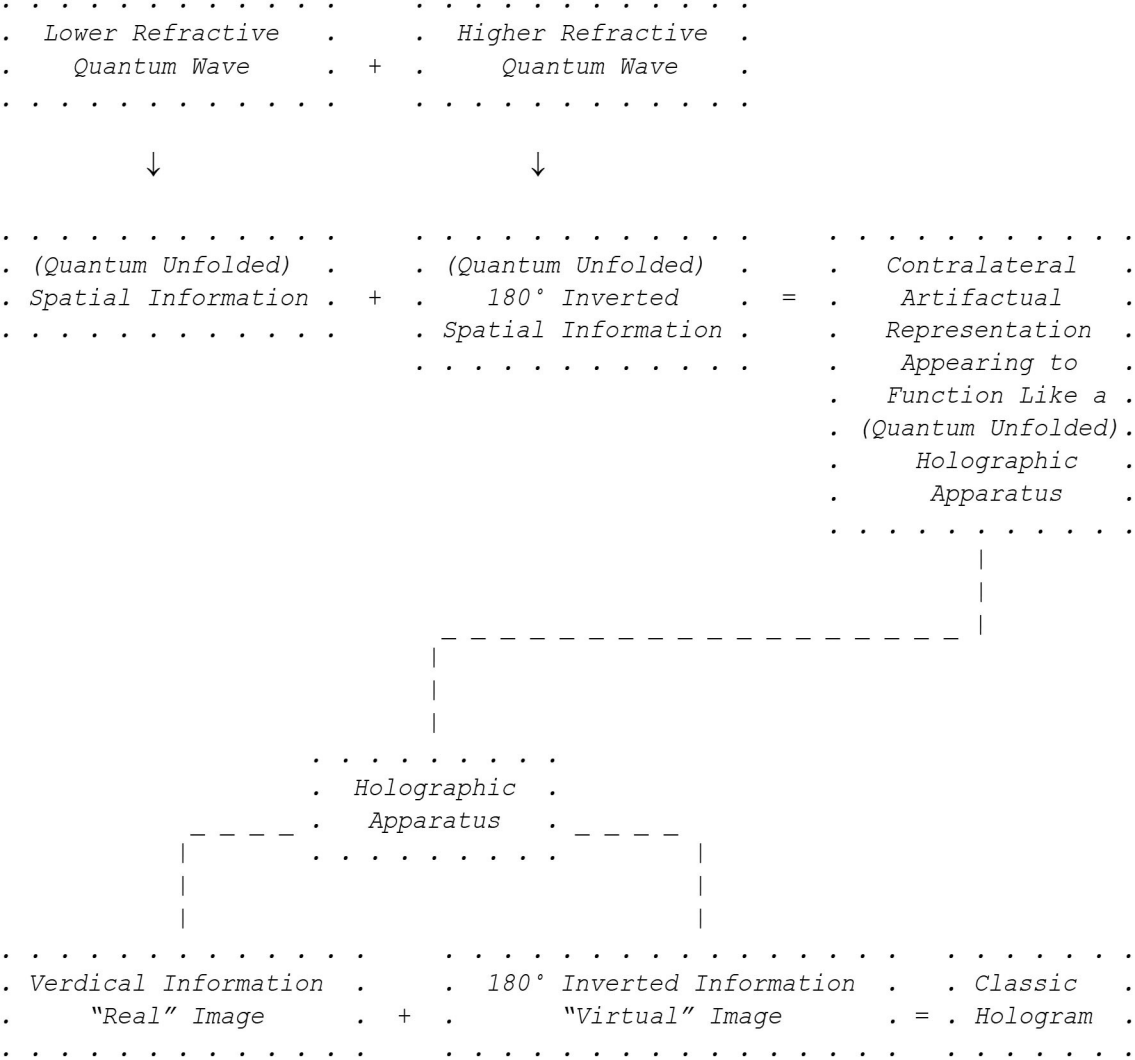
Schematic outline of the working hypothesis presented here that the contralateral organization of the human nervous system appears to function like a quantum unfolded holographic apparatus by appearing to invert and reverse quantum unfolded visual and non-visual spatial information (as proposed in the preceding paragraph).

In support of the working hypothesis, a 180° up-down inversion and right-left reversal of spatial information is shown for the three-dimensional representation of the contralateral organization, together with veridical information, that would appear in functional agreement with how a classic holographic apparatus would similarly appear to operate, which also appears to utilize both 180° up-down inverted and right-left reversed information (“virtual” image) and veridical information (“real” image). A quantum wave can also become right-left reversed as a result of being 180° inverted when moving from a lower to a higher refractive index. A quantum wave at a lower refractive index does not undergo 180° inversion. 180° inversion is a general property of waves: water, radio, light, electromagnetic, quantum, etc. (Bleany and Bleany, 2013). As shown in Figure 1, both types of quantum waves would create an interference pattern and act together two-dimensionally to unfold information that would generate the three-dimensional holographic world we who are “observers” experience, in a way consistent with how both a classic holographic apparatus and the three-dimensional holographic contralateral organization would appear to operate.

It is speculated here that we the observers exist at the two-dimensional level and are encoded to perceive two-dimensional information as unfolded three-dimensional visual and non-visual spatial appearances, so as to provide a working context for new encoding/folding (i.e., conscious change) within the two-dimensional quantum digital system. An observer would be a form of artificial intelligence because an observer is digitally “encoded” for participation and this would, of course, argue for the observer being the product of intelligent design. It seems oxymoronic that any sort of evolutionary process could independently arise within a digital system that would arrive at artificial intelligence. Moreover, there is a scarcity of transitional fossils within the three-dimensionally appearing world that would support a long, gradual evolution. However, the three-dimensional fossil record shows a sudden emergence of many new cell types that appear fully functional across many phyla during the early Cambrian Period (Meyer, 2014). This provides an argument for what may be programmable design within the two-dimensional quantum digital system that has been hypothesized.

Various methodologies and research observations reported elsewhere are reviewed and interpreted in this article that, when considered together, provide support for the working hypothesis. What follows is divided into six sections. The first section describes the classic holographic method and characteristics of image formation contained by a holograph. The double-slit experiment is described in the second section and its quantum functioning compared with the basic holographic operation. The next three sections review evidence for inversion and reversal of visual and non-visual spatial information that is shown by the human nervous system. Finally, a discussion of the evidence for the contralateral organization appearing to operate like a holographic operation is provided. Thus far this article has been written using a two-dimensional perspective, under the terms of which the causes and effects we perceive to be going on in three dimensions are happening in a two-dimensional quantum plane. The next five sections are written using a three-dimensional perspective because the materials reviewed are written by authors who use terms that describe causes and effects that would occur in a three-dimensional world that is not holographic. This article's final section (Further Discussion) is written to the greatest extent possible using a two-dimensional perspective.

## Holography and inversion and reversal of visual spatial information

Coherence is one of the most important concepts in optics and is strongly related to the property of light waves to produce stationary (both temporally and spatially) interference patterns for creating a hologram. Two light wave sources are “coherent” if their frequency and waveform are identical. The general method of beam combining for producing a hologram relies on this mutual coherence. Because separate light sources cannot maintain coherence for periods long enough to record a holographic interference pattern, a single light source is used in conjunction with a pair of mirrors to accomplish this. Since a laser generates very pure coherent light in this shared context, it is especially good at producing stationary wave interference patterns. A classic hologram is created when a single laser beam is split into two separate light beams. The “beam splitter” used is a very high grade half-silvered glass (i.e., a one-way mirror). When properly angled, half the laser light is transmitted through the glass (first light beam) and is bounced off an object that is to be photographed, becoming the “real” image. The other half of the laser light is reflected by the mirror and then by a second mirror, which together invert and right-left reverse the light as part of a 180° rotation. This second light beam then intersects with the light from the first beam creating the wave interference pattern for the “virtual” image. When the two beams intersect, they appear on a piece of photographic film as a peculiar set of concentric circles that represent the interference pattern. Because corresponding points on the real and virtual images are located at equal distance from the plane of the hologram, the virtual image appears “pseudoscopic” (i.e., its depth appears inverted with its three-dimensional appearance in reverse). As an illustration, buildings appear to look depth-wise like swimming pools in aerial photography, and swimming pools look like buildings. The real image is “orthoscopic” because it appears correct and in normal proportions. When a light beam from the same kind of laser is shined through the photographic film a three-dimensional image of the object is produced. This image exhibits all the effects of perspective and depth of focus that characterized the original photographed object (Hariharan, 2002; Saxby and Zacharovas, 2016).

## The double-slit experiment and inversion and reversal in superposition

The actions of the double-slit experiment demonstrate quantum wave-like interference patterns. In the classic double-slit experiment, a stream of photons (or electrons or any atomic-sized objects) are shot at a screen with two tiny slits in it. On the back side of the screen, a photographic plate or highly sensitive video camera records where each photon arrives. If one of the slits is closed, a smooth distribution of photons will appear with the peak intensity directly opposite of the open slit, which would be expected if the photons were individual particles. However, when both slits are open, a different pattern appears: an interference pattern of bright and dark bands on the screen, which is consistent with the photons being waves (Lederman and Hill, 2001).

A simpler version of the double-slit experiment uses a Mach-Zender interferometer; whereby, photons can only travel via two paths that each have one discrete photodetector. The apparatus utilizes a beam-splitter with mirrors (in a way that resembles how a hologram is created) for the purpose of causing a quantum particle to be “entangled in superposition” between the two possible paths. When entangled in superposition, the position of a single particle is spread out as two particles over space (i.e., as a wave) which always spin sideways opposite each other, clockwise and counterclockwise; one having right-left reversed spin, as the result of being 180° inverted relative to the other particle (Vlatko, 2006).

A Michelson interferometer also uses a beam splitter with mirrors and is involved in double-slit experiments. Subject concentration effects of meditating and not meditating on a Michelson interferometer during a double-slit experiment showed a significant relationship for directing attention to the experimental apparatus, which was interpreted to provide support for the conscious observer hypothesis (Radin et al., 2021).

## Perceptual plasticity in the human nervous system

It is remarkable that most of the ascending and descending sensorimotor communication tracts cross in either the medulla oblongata or the spinal cord. As a result of this crossing, nearly all of the sensory impulses received on one side of the body are perceived by the contralateral cortex. Moreover, fibers originating from one side of the cortex control primarily, if not entirely, muscle movements on the contralateral side of the body (Tortora and Derrickson, 2020).

Various research observations related to the contralateral organization were previously reviewed and interpreted by this author (Zukauskis, 2012) and are again presented here. It was proposed that, in response to the contralateral organization, conscious awareness is normally an illusory (right-left reversed) visual, auditory, tactile, and proprioceptive perception of the external three-dimensional world. This idea arises from perceptual plasticity investigations that require subjects to wear, over several consecutive days, distortion goggles (optical devices containing lenses, prisms, or mirrors) that invert or reverse the retinal image. Importantly, the brain does not change the cortical representation of the goggle retinal image to agree with the spatial properties that would otherwise be encoded from the normal retinal image. There is no compensatory change in visual perception with adaptation to distortion goggles. There is widespread agreement that adaptation to distortion goggles is entirely kinesthetic and proprioceptive (Harris, 1980; Linden et al., 1999; Richter et al., 2002). Kinesthesia refers here to the conscious perception of body position that is brought about by the directional movements and muscle tensions of the limbs and other body parts. Proprioception refers here to the unconscious perception of movement and spatial orientation arising from stimuli within the body itself. The subjects' kinesthetic and proprioceptive perceptions of their arms, legs, and entire bodies feel reversed (and in agreement with what they see) after wearing right-left reversing goggles for an extensive period of time. Adaptation to the goggles becomes so complete subjects have stopped noticing that the letters and words still look backwards. The subjects' position sense also accurately adapts kinesthetically and proprioceptively to up-down inverting goggles (Harris, 1980). It has been noted that adaptation to up-down inverting goggles takes considerably fewer days than with right-left reversing goggles (Livingston, 1978).

Right-left reversing goggles might take considerably more days to adapt to because adaptation to them may require further trial-and-error muscle movements that use real (i.e., pre-goggle) sensorimotor memories, which would have to be distinguished from visual and non-visual memory reversals ordinarily stored in the brain in a right-left way for each real memory experienced. Which would be consistent with how right-left reversed virtual images are stored together with real images in a holographic interference pattern, as was previously described in the holography section. Evidence for right-left, visual and non-visual memory reversals being ordinarily stored in the brain is reviewed later in this article. Up-down goggle adaption would presumably favor the use of real, proprioceptively learned sensorimotor memories cued by gravity and sunlight that involve far less spatial abstraction than when distinguishing real, sensorimotor memories from right-left reversed sensorimotor memories.

When subject's view the world normally, reversed imagery appears on their retinas in both the up-down and right-left directions. An example of a line of printed words that appears upside-down and right-left reversed by a normal up-down 180° rotation on the retina, or one made by a holographic apparatus, is:

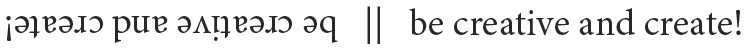

Different from this, up-down goggles reverse in the up-down direction only due to a mirror-image reversal. Likewise, the spatial properties produced by right-left reversing goggles result from mirror-image reversal of the visual field and are not equivalent to those that are right-left reversed by a normal up-down 180° lens rotation or a holographic apparatus. An ordinary mirror does not reverse imagery right to left, it reverses (i.e., reflects) imagery front to back. Nonetheless, subjects' who wear right-left reversing goggles report their kinesthetic and proprioceptive perceptions feel right-left reversed after adaptation. An example of a line of printed words that is mirror-image reversed (without an up-down 180° rotation) by right-left reversing goggles is:

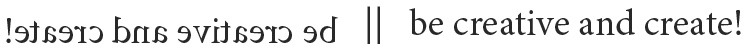

## The contralateral organization of the human nervous system and inversion and reversal of visual and non-visual spatial information

The lens of each eye, without distortion goggles, normally produces an up-down inversion that contains a right-left reversal upon each retina, appearing as an image on film in a camera would. When the eyes are fixated, the right visual field projects onto the nasal half of the retina of the right eye and the temporal half of the retina of the left eye. The axons from both of these hemiretinas terminate in the left occipital lobe so it processes the right visual field, and vice versa the right occipital lobe processes the left visual field. In conjunction with this, the upper half of the right visual field (or upper-right quadrant) is processed in the left occipital area below the calcarine sulcus and the lower half of the right visual field (or lower-right quadrant) is processed in the left occipital area above the calcarine sulcus and vice versa for the right occipital lobe and left visual field (Tortora and Derrickson, 2020). This outline implies the cortical representation of the field of vision is normally inverted and reversed (i.e., homologous with the normal retinal image). Given the demonstrated plasticity of the nervous system for goggle adaptation, which is entirely kinesthetic and proprioceptive, it is presumed here that the brain does not change the cortical representation of the normal up-down inversion that contains a right-left reversal produced by the lens. It would seem unnecessary and inefficient to do so. Loosemore (2011) has theorized that the 180° optical inversion by the lens of each eye might explain the contralateral organization of the nervous system.

When the retinal image is right-left reversed by distortion goggles, not only does body position sense adapt to it, but spatial location for sound also shifts in the direction of the altered visual sense. Visual capture of sound location occurs instantly, with little or no notice of intersensory discrepancy. This phenomenon is known as the ventriloquism effect (Howard and Templeton, 1966). However, this spatial plasticity is not considered to be adaptation because there is no change in auditory perception lasting beyond the visual exposure period (Welch, 1978). The explanation being proposed here for this phenomenon links with how the pathway for each ear is primarily connected to the contralateral cortex (Tortora and Derrickson, 2020).

## Right-left reversal in the human nervous system without distortion goggles

Two well-known examples of reversal of visual perception, without distortion goggles, are stroboscopic presentations and spoked wheels turning in video frames which both at times appear to sporadically alternate clockwise and counterclockwise (i.e., becoming inverted and having right-left reversed spin). Spoked-wheel illusions also occur in continuous light (i.e., in the absence of intermittent illumination). A well-known example of right-left reversal of motor movement, without distortion goggles, is mirror writing (writing backwards) brought about by brain injury (Feinberg and Jones, 1985). According to Hécaen and de Ajuriaguerra (1964), 80% or more of most normal adults discover they can easily write backward with the non-preferred hand when asked to. Kushnir et al. (2013) found most right-handed people are able to mirror write with the left hand with minimal practice.

Evidence for the existence of a right-left reversal process being involved with visual memory is provided by a classic experiment performed by Standing et al. (1970). They showed people 2,560 pictures of ordinary scenery for 10 seconds each, and when tested after 3 days, the subjects were able to achieve a high level of identification. What is noteworthy about their experiment is that right-left reversed photographic images, which had not been shown, were identified as often as the originals. In a similar vein, Rock (1973) describes an unpublished experiment by Olshansky in which people were shown novel shapes and then 2 min later tested for identification. Identification for right-left reversed images was almost that of the originals, but not similarly mistaken when the original images were inverted upside-down. False memory for inverted images would not be expected because memory for originally seen images would include up-down spatial information cued by gravity for proprioception. Because there are no apparent environmental cues such as gravity for distinguishing right and left, false memory for right-left reversed images would be expected if right-left, visual memory reversals are stored in the brain for each original image seen——akin to right-left reversed virtual images being stored together with real images in a holographic interference pattern, as was previously described in the holography section. Perhaps analogous to this, non-dyslexic children often find it difficult to establish the correct orientations for the letters of the alphabet when first learning to read and write. Their letters are sometimes written backwards. Most noticeably, they fail to distinguish the mirror-image lowercase b from d and p from q. They rarely make d-p, b-q, d-q, or b-p up-down reversals (Davidson, 1935).

## Further discussion

The adaptive advantage of the contralateral organization of the human nervous system is unknown (Rodieck, 1998). And it is not known why the human nervous system demonstrates potentials for up-down inversion and right-left reversal associated with body position sense and right-left reversal of sound. It is presumed here that the same things appear to happen for a normal operation of kinesthetic and proprioceptive mechanisms as shown with distortion goggles——due to the lens of each eye normally showing an up-down inversion that contains a right-left reversal upon each retina. Because potentials are shown for distortion goggle adaptation, it is presumed here they would be integral for a normal operation of kinesthetic and proprioceptive mechanisms.

It is important to note the same functional topological pattern (contralateral organization) is shown for what would be visually experienced as for what would be non-visually experienced. Suppose, then, one links the kinesthetic right-left reversal potential with the sensorimotor tracts crossing, considers the pathway for each ear is primarily connected to the contralateral cortex, and considers the evidence the cortical visual representation is homologous with the normally inverted and right-left reversed retinal image. These considerations carry the presumption that any intersensory organization capable of producing concurrent multimodal perceptual information about the same spatial event, presupposes separate underlying neurophysiologic mechanisms synchronized in a directionally related point-to-point correspondence. Cortical mapping of non-visual with visual information would seem most efficient in this way. It was, therefore, theorized in the (Zukauskis, 2012) article that the sensory tracts crossing would provide for a right-left reversal of non-visual spatial properties that would become coded in concordance with the inverted and right-left reversed spatial properties of the retino-cortical representation. Moreover, the motor tracts crossing would bring about orientation and movement on each side of the body that veridically corresponds to an external three-dimensionally appearing reality, although directionally reversed in relation to what the person would consciously see, hear, and feel. The normally appearing kinesthetic and proprioceptive actions would thus be directionally opposite of those observed for right-left reversal adaptation brought about by distortion goggles——quite logically presuming the same perceptual motor model and contralateral pathways would be followed in both instances. The obvious implication of this would be a normal body position sense dependent upon an illusory (right-left reversed) visual, auditory, tactile, and proprioceptive sensory representation of an external three-dimensionally appearing reality. People are not aware that their everyday visual and non-visual perceptions of their external three-dimensionally appearing reality are *all normally right-left reversed* (thus believing they are writing or holding a fork with their right hand when they are actually doing so with their left hand or turning left when driving a car when they are actually turning right); because the motor tracts crossing would right-left reverse the brain's motoric response pattern for their normally illusory perceptions so as to accurately match orientation and movement with external, three-dimensionally appearing reality. The same form of perceptual processing would be expected to normally occur as observed with right-left reversing goggles——given that the same underlying neurophysiologic mechanisms would most arguably be used in the same way or a similar fashion by both.

Key to an understanding of the working hypothesis that the contralateral organization of the human nervous system appears to function like a quantum unfolded holographic apparatus by appearing to invert and reverse quantum unfolded visual and non-visual spatial information, distortion goggles demonstrate human nervous system potentials for up-down inversion and right-left reversal associated with body position sense and right-left reversal of sound. Longstanding evidence for right-left reversal of visual and non-visual perception and visual and non-visual memory appearing in the nervous system without distortion goggles is presented in this article, some of which is fairly old but entirely relevant. Corresponding with the up-down inversion and right-left reversal of spatial information that is shown for the contralateral organization, a classic holographic apparatus also appears to operate using up-down inverted and right-left reversed spatial information. Moreover, a Mach-Zender interferometer used to produce a simplified version of the double-slit experiment, appears to operate like a holographic apparatus using a beam-splitter and mirrors to show a quantum particle to be entangled in a superposition that has up-down inverted and right-left reversed properties. *All of which suggests, up-down inversion and right-left reversal processes are fundamentally involved with and central to image formation for holographic devices, the human nervous system, and quantum waves*. Furthermore, this same unmistakable pattern that is present for contralateral organization, holographic, and quantum operations is also present for alternating current and other physical phenomena. In normal AC power, the current periodically alternates between the up and down and left-right and right-left directions in a sine-wave pattern (Bhargava et al., 2013).

Scientists talk about “folding space” for time-efficient interstellar travel. Given the laws that rule our three-dimensionally appearing experience, future interstellar spacecraft could not exceed the speed of light. However, according to the holographic principle, space *is* already folded like everything else in the two-dimensional plane. Discovering how to obtain and apply knowledge of two-dimensional folded space, for time-efficient interstellar travel, might be gained from creating artificial intelligence that has infinite computational capacity for doing Quantum Logic. Such a discovery would seemingly prove the holographic principle, that everything we see going on around us in our three-dimensional experience is occurring in a two-dimensional plane. As previously mentioned, anomalies such as future interstellar spacecraft that exceed the speed of light show up that ignore laws that appear to rule our three-dimensional reality.

## Data availability statement

The original contributions presented in the study are included in the article/supplementary material, further inquiries can be directed to the corresponding author.

## Author contributions

The author confirms being the sole contributor of this work and has approved it for publication.
